# The influence of primary care quality on nursing home admissions in a multimorbid population with and without dementia in Germany: a retrospective cohort study using health insurance claims data

**DOI:** 10.1186/s12877-021-02731-8

**Published:** 2022-01-14

**Authors:** Kathrin Seibert, Susanne Stiefler, Dominik Domhoff, Karin Wolf-Ostermann, Dirk Peschke

**Affiliations:** 1 Faculty 11: Human and Health Sciences, Institute for Public Health and Nursing Research, University of Bremen, Grazer Str. 4, 28359 Bremen, Germany; 2 High Profile Area Health Sciences, University of Bremen, Bremen, Germany; 3 Department of Applied Health Sciences, Hochschule für Gesundheit (University of Applied Sciences), Bochum, Germany

**Keywords:** Health claims data, Dementia, Quality, Multi-morbidity, Nursing home

## Abstract

**Background:**

Multimorbidity poses a challenge for high quality primary care provision for nursing care-dependent people with (PWD) and without (PWOD) dementia. Evidence on the association of primary care quality of multimorbid PWD and PWOD with the event of a nursing home admission (NHA) is missing. This study aimed to investigate the contribution of individual quality of primary care for chronic diseases in multimorbid care-dependent PWD and PWOD on the duration of ongoing residence at home before the occurrence of NHA.

**Methods:**

We conducted a retrospective cohort study among elderly care-dependent PWD and PWOD in Germany for six combinations of chronic diseases using statutory health insurance claims data (2007–2016). Primary care quality was measured by 21 process and outcome indicators for hypertension, diabetes, depression, chronic obstructive pulmonary disease and heart failure. The primary outcome was time to NHA after initial onset of care-dependency. Multivariable Cox proportional hazard models were used to compare the time-to-event between PWD and PWOD.

**Results:**

Among 5876 PWD and 12,837 PWOD 5130 NHA occurred. With the highest proportion of NHA for PWD with hypertension and depression and for PWOD with hypertension, diabetes and depression. Average duration until NHA ranged from 6.5 to 8.9 quarters for PWD and from 9.6 to 13.5 quarters for PWOD. Adjusted analyses show consistent associations of the quality of diabetes care with the duration of remaining in one’s own home regardless of the presence of dementia. Process indicators assessing guideline-fidelity are associated with remaining in one’s home longer, while indicators assessing complications, such as emergency inpatient treatment (HR = 2.67, 95% CI 1.99–3.60 PWD; HR = 2.81, 95% CI 2.28–3.47 PWOD) or lower-limb amputation (HR = 3.10, 95% CI 1.78–5.55 PWD; HR = 2.81, 95% CI 1.94–4.08 PWOD) in PWD and PWOD with hypertension and diabetes, increase the risk of NHA.

**Conclusions:**

The quality of primary care provided to care-dependent multimorbid PWD and POWD, influences the time individuals spend living in their own homes after onset of care-dependency before a NHA. Health care professionals should consider possibilities and barriers of guideline-based, coordinated care for multimorbid care-dependent people. Further research on quality indicator sets that acknowledge the complexity of care for multimorbid elderly populations is needed.

**Supplementary Information:**

The online version contains supplementary material available at 10.1186/s12877-021-02731-8.

## Background

Dementia is an increasing international syndrome that affects around 50 million people worldwide and that contributes eminently to disability and dependency among elderly people [1]. By the end of 2018, about 1.6 million people aged 65 years or above were living with a dementia diagnosis in Germany, corresponding to 6.8% of the overall population in this age group [2]. Dementia and other cognitive impairments are predictors for nursing home admission (NHA) in the earlier or later life-course [3] and people with dementia (PWD) are specifically dependent on others to achieve adequate access to primary care and participation in treatment processes [4]. In this context, multimorbidity, which can be defined as the occurrence of two or more chronic diseases or conditions [5], poses an additional challenge for primary care delivery for PWD [6, 7]. Multimorbidity is highly prevalent in PWD [6, 8] as well as in elderly and care-dependent populations in general [4, 9–11]. For PWD, utilization of primary care physicians and unplanned hospital admissions increase in relation to the number of additional diseases [6]. This finding is in line with research on the association of the number of diseases and the frequency of medical care services utilization in populations of people without dementia (PWOD) [9]. In addition, dementia may complicate the management of comorbid conditions which in return may affect the progression of dementia [6, 12, 13].

Evidence on organizing primary care for PWD and PWOD affected by multmorbidity is limited and the use of guidelines that are of a mono-morbid nature can facilitate adverse events such as drug interactions, contradictory treatment strategies or harmful polypharmacy [4, 9]. A leading German treatment guideline for multimorbidity [9] acknowledges the heterogeneity of the patient population and therefore does not incorporate specific diseases but focuses on care processes and treatment decisions in primary care which should aim at improving multimorbid patients’ quality of life and functional abilities and at strengthening self-management strategies [9]. The higher the number of health care providers involved in the treatment of people affected by mutlimorbidity, the more complex the processes for sharing and synchronizing information regarding diagnostic or therapeutic decisions become [9]. The chronic care model (CCM) provides a theoretical reference and reasoning framework for the cooperative-collaborative design and organization of care delivery [14, 15]. As an ideal-type model to improve outcomes in chronic illness care the CCM highlights cooperative, proactive health-care practice teams that implement team-based, planned and shared care next to an active role of patients [14, 15]. Patient-centered care for multimorbid populations implies sufficient time for communicating and negotiating decisions and sufficient communicative abilities of health care providers and patients [9]. The latter posing another challenge for the treatment of PWD. The CCM makes a case for decision-support of health care providers as well as for the utilization of clinical information systems that can be used to measure and improve quality of care of and to control care processes [14, 15]. The active co-responsible role assigned to patients in the CCM which can also be found in the Germany statutory health insurance (SHI) calls for the engagement in quality assurance activities for PWD and care-dependent persons in general on the level of single providers, practice teams, provider networks and the health system itself. The analysis of SHI claims data to gain information on health care quality on a population level can support health care providers in evaluating or identifying potential gaps in the care for multimorbid patients who are limited in their abilities to take on an active role in the treatment-process.

Health care quality indicators (QI) are being utilized worldwide as a means to assess and improve quality of primary health care [16, 17]. Following Donabedians’ model for assessing health care quality [18], next to QI that relate to the structure of care, a wide selection of QI assessing processes and outcomes of care is available internationally [17]. To our knowledge, there are no comprehensive indicator sets for assessing the quality of primary care provided to care-dependent multimorbid PWD and PWOD that take combinations or patterns of diseases into account that have been reported to be prevalent in these populations [8, 19]. Previous studies have shown effects for selected processes or outcomes of primary care on the event of NHA. For example, sustained systolic blood pressure control is associated with a lower risk of long-term NHA [20] and targeted interventions to improve medication management for community-dwelling frail elders may reduce NHA [21], and QI have been shown to attribute for 40% of variations for the proportion of NHA and 49% for the time until NHA in an analysis of informal provider networks in Germany [22]. While the quality of diabetes care processes has been reported to improve for PWD after NHA in comparison to the year before NHA [23], pointing to quality deficits prior to NHA, empirical evidence on the association of primary care quality of care dependent multimorbid PWD and PWOD with the occurrence of NHA is missing. A broad spectrum of chronic diseases is prevalent in residents of nursing homes [13, 24, 25] for which a targeted alignment of the quality of primary care delivered prior to NHA may contribute to preventing or delaying NHA. Furthermore, evidence on aspects of primary care quality of multimorbid PWD and PWOD can indicate needs and opportunities for care improvement. This background, in combination with the facts that people want to stay in their own homes in old age as long as possible [26, 27], and that the German long term care insurance prioritizes ambulatory home care over nursing home care, constitutes the research interest of this study.

We aim to investigate the contribution of individual quality of primary care for chronic diseases in multimorbid care dependent PWD and PWOD on the duration of ongoing residence at home before the occurrence of NHA. We assume that the better the individual quality of care, the longer the time spent in the people’s own home. Furthermore, group differences in the quality of care between multimorbid PWD and PWOD are of interest.

## Methods

This retrospective cohort study with an observation period of 10 years (2007 to 2016) uses anonymized SHI claims data from persons aged 65 years or older, insured with one of the 11 AOK SHI funds in Germany. We follow the STROSA 2 reporting standard [28], specifically developed for analyses of secondary data and their specific requirements for the German health care system.

### Data sources

With more than 27 million insurees, the AOK SHI funds insures about a third of all people insured under the German SHI system and has a share of almost 50% among all care-dependent people in Germany. The analyzed SHI claims data comprises of the entire individual in- and outpatient health care history of the insurees, including all diagnoses, prescribed and provided medications, medical procedures, rehabilitation services and physical, occupational, speech and language therapy, podology, level of care and place of residence (nursing home or community-dwelling) as well as personal data including age, sex, and federal state of residence. In order to include information on functional and cognitive impairments and social integration as well as on the diagnosis leading to care-dependency which is not included in the SHI claims data, anonymized data from the eligibility assessment for German long-term care provided by the German Medical Service of the Health Funds (MDK) were merged with the SHI claims data using a three-stage deterministic data linkage at the person level based on the variables MDK region, gender, date of birth, and level of care. For persons without a unique match, the diagnosis leading to care-dependency from the MDK data and the outpatient and inpatient diagnoses from the SHI claims data were added as linkage variables, first with three-digit diagnosis coding, then with end-digit diagnosis coding according to the International Classification of Diseases 10. Revision German Modification (ICD-10-GM). Individuals whose records could not be unambiguously matched even in the third linkage step were excluded from the analyses. This applied to 69,861 records in the SHI claims data and 139,995 records in the MDK data.

### Sample and sample size

The observation cohort includes all AOK insurees who were at least 65 years old and who became initially care dependent in 2006 and did not live in a nursing home in that year. The study population was divided into PWD and PWOD according to the documentation of a dementia diagnosis (ICD-10-GM code F00-F03) in 2006. In addition, a diagnosis of at least two other chronic diseases had to be documented in at least two quarters in 2006. All individuals were followed until the quarter of their exit from the study due to NHA or death or end of the observation period at the end of 2016. The analysed cohort included 18,713 individuals with a total of 267,182 person-quarters at risk and 5130 target events (NHA). Due to the study design as a retrospective and exploratory cohort study, no prior sample size calculation was performed.

### Data Protection and ethics approval

SHI claims Data were provided by the AOK Federal Association as the data holder on behalf of all AOK insurance companies. The long-term care assessment data were provided by the Medical Service of the National Association of Health Insurance Funds (MDS). Both data holders anonymised the data to preclude the identification of individuals while retaining the possibility to observe individuals longitudinally and between sectors of health care. The data protection officer of the AOK Federal Association approved an operation procedure for data protection, restricting usage of the data for the study’s purpose. The need for an ethics approval and informed consent is waived by the German federal regulation in §§ 67b & 75 German Social Code, Book X. The German Social Code regulates the usage of social data for the purpose of research. Use of the data without the informed consent of the persons included in this study is permitted by German law, as only anonymous data were used.

### Construction of disease groups

**Fig. 1:**
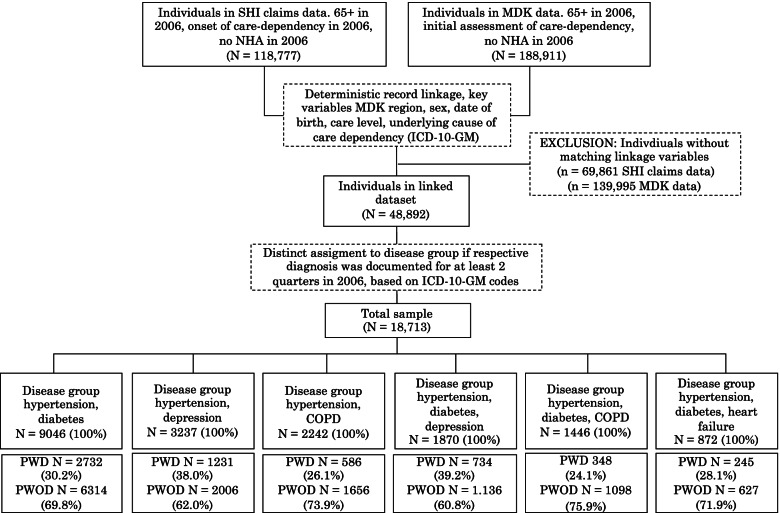
Flow chart for study population. SHI = Social health insurance, MDK = Medical Service of the Health Funds, NHA = Nursing home admission, IC-10-GM = International Classification of Diseases 10. Revision German Modification, COPD = Chronic obstructive pulmonary disease, PWD = People with dementia, PWOD = People without dementia

As there is no index for multimorbidity available that allows to establish an individual reference to relevant QI, a data-driven approach was used to identify patterns of combinations of diseases for which common quality indicators were available. For the identification and construction of disease groups, combinations of diseases were taken into account that were documented for at least 200 PWD in at least two quarters in 2006. This condition applied to six constellations of chronic diseases (see Fig. 1). The individual diseases were defined using the ICD-10-GM codes for asthma (J45), chronic obstructive pulmonary disease (COPD, J43, J44), hypertension (I10-I13, I15) heart failure (I50.1), type 2 diabetes mellitus (diabetes, E11) and depression (F32, F33, F34.1) and individuals were distinctively assigned to one disease group.

The data were analyzed at the person level. The target event is the first occurrence of NHA. Individual characteristics as well as individual QI scores, were included as exposure and control variables. Additional file 1 shows all independent variables included in the study and their specifications, which include the following variables.

### Variables

Individual characteristics such as age in years, gender, and level of care, as well as other characteristics described in the literature as predictors of NHA [29] were considered for risk adjustment. These characteristics include the presence of functional and cognitive impairments and the direct social network (living alone or with another person in the household). Furthermore, the Charlson comorbidity index with all ICD-10-GM diagnoses reported by Quan et al. [30] and the presence of osteoarthritis (ICD-10-GM M15-M19) and osteoporosis (ICD-10-GM M80.0) were included, as the latter two diagnoses also occurred in PWD in combination with the other conditions when the disease groups were constructed, but applied to fewer than 200 individuals.

Quality of care for chronic diseases was assessed by 21 QI, which were selected in a stepwise procedure. A literature-based selection of QI [31] was discussed for clinical and practical relevance, data availability, and coding quality with external medical expertise. Numerator and denominator definitions were then established. Operationalization was based on ICD-10-GM diagnoses, operation and procedure codes, fee schedule item numbers, and drug substance groups. The QI relate to the care of the six disease groups and predominantly include process indicators that target guideline-appropriate prescribing behaviour or recommended diagnostics. Undesired events that may occur as an endpoint of the outpatient care process were included as outcome indicators. These include emergency inpatient treatment for diabetes, COPD, or heart failure, or lower-limb amputation in individuals with diabetes.

### Data processing and analysis

QI values were calculated quarterly at the individual level and checked for plausibility by comparison with reference values derived from the literature. The first quarter of 2007 was defined as the baseline quarter, since a person in the observation cohort could only move into a nursing home from this quarter onward by definition. The fourth quarter of 2016 represented the end of the observation period. Characteristics of the study population were analyzed by descriptive, univariate analyses. The impact of individual quality of care on the event of NHA was determined using time-to-event analysis. Multivariable Cox regression models were calculated to account for the temporal relationship (temporal proximity) between time-varying variable characteristics and event occurrence [32–34]. We plotted Schoenfeld residuals [33] against event time to graphically assess the proportionality assumption for each model which held true for the majority of covariables. Throughout the disease groups, a consistent violation of the proportionality assumption occurred for the covariable expressing the number of functional impairments derived from the MDK data that was used for adjusting the model. One model was calculated per disease group and QI separately for PWD and PWOD. The results, expressed as hazard ratios (HR), indicate an increased risk of NHA or a shorter duration of stay in a persons’ own home until NHA if the HR is > 1. An HR of < 1 indicates a reduction in risk of NHA or a longer length of stay in the home. We further examined the combined influence of the variables that were used to adjust the models containing the QI on NHA by calculating one model per disease group and separately for PWD and PWOD. The significance level chosen was alpha = 0.05. Data processing and statistical analysis were performed using the SAS® version 9.4 software package.

## Results

The study includes a total of 18,713 individuals (5876 PWD and 12,837 PWOD). Figure 1 shows a flow chart of the allocation and case numbers in the disease groups for PWD and PWOD. In each disease group, PWOD make up at least 60% of the persons assigned to it and PWD less than 40%.

Table 1 shows the baseline characteristics of the study population. During the 10-year follow up, the median length of observation was 11.9 quarters for PWD and 15.6 quarters for PWOD. Overall, 63.8% (*n* = 11,941) of the individuals in both groups were female. PWD were on average 2 years older than PWOD at 81.7 years. For the distribution of levels of care, both groups showed the highest proportions for care level 1 (65.6%, *n* = 3854 PWD and 75.7%, *n* = 9721 PWOD) while 30.0% of PWD (*n* = 1762) and 21.0% of PWOD (*n* = 2694) were assigned to care level 2. Both groups included small proportions of individuals without a care level who had been downgraded in the observation period after being classified as initially needing care in 2006. On average, almost equal proportions of individuals in both groups lived alone (43.0% PWD versus 43.4% PWOD). The mean number of cognitive impairments (sum of documented anomalies in orientation, drive/motivation, mood, memory, day-night rhythm, perception and thinking, communication/language, situational adaptation, and participation in social aspects of life) was 4.9 for PWD and 2.3 for PWOD. The mean number of functional impairments was similar in both groups. PWOD were prescribed an average of 7.2 different medications compared with 6.4 different medications for PWD, and the proportion of individuals receiving a medication potentially inappropriate for the elderly was 3.4 percentage points higher for PWOD than for PWD, at 32.9%. Selected chronic disease diagnoses also showed similar distributions between the two groups. Differences of 5% or more are evident for the proportion of individuals with COPD (20.2% PWOD versus 14.7% PWD) and depression (28.9% PWD versus 23.4% PWOD). Additional file 2 shows the distribution of QI measures in the baseline quarter, which predominantly showed similar values for PWD and PWOD.

**Table 1:** Baseline characteristics, 2007, 1st quarter. Absolute and relative frequencies unless otherwise indicated

	People with dementia (*n* = 5876)	People without dementia (*n* = 12,837)	Total (*n* = 18,713)
Time under risk in quarters, mean, (SD)	11.9 (10.8)	15.6 (12.7)	14.4 (12.3)
Age in years, mean, (SD)	81.7 (6.9)	79.6 (7.3)	80.3 (7.3)
Female gender	3762 (64.0)	8179 (63.7)	11,941 (63.8)
Level of care			
none	31 (0.5)	110 (0.9)	141 (0.8)
I	3854 (65.6)	9721 (75.7)	13,575 (72.5)
II	1762 (30.0)	2694 (21.0)	4456 (23.8)
III	229 (3.9)	312 (2.4)	541 (2.9)
Social network			
Living alone	2524 (43.0)	5565 (43.4)	8089 (43.2)
Number of cognitive impairments, mean (SD)	4.9 (3.4)	2.3 (2.9)	3.2 (3.3)
Number of functional impairments, mean (SD)	3.6 (0.8)	3.5 (0.8)	3.5 (0.8)
Number of prescribed medications, mean (SD)	6.4 (3.5)	7.2 (3.9)	6.9 (3.8)
Individuals with potentially inappropriate medication in the elderly	1735 (29.5)	4217 (32.9)	5952 (31.8)
Diagnoses			
Asthma	31 (0.5)	118 (0.9)	149 (0.8)
COPD	864 (14.7)	2591 (20.2)	3455 (18.5)
Hypertension	5271 (89.7)	11,699 (91.1)	16,970 (90.7)
Heart failure	436 (7.4)	1050 (8.2)	1486 (7.9)
Coronary heart disease	2552 (43.4)	5944 (46.3)	8496 (45.4)
Diabetes mellitus type 2	3616 (61.5)	8337 (65.0)	11,953 (63.9)
Osteoarthritis	1940 (33.0)	4729 (36.8)	6669 (35.6)
Osteoporosis	908 (15.5)	2141 (16.7)	3049 (16.3)
Depression	1699 (28.9)	3004 (23.4)	4703 (25.1)

*SD* Standard Deviation, *COPD* chronic obstructive pulmonary disease

During the observation period, 3505 (59.6%) PWD and 8607 (67.0%) PWOD died while living at home, and 2120 (36.0%) PWD and 3010 (23.4%) PWOD were admitted to a nursing home. The numbers of NHA per disease group as well as the average time in quarters between the onset of care-dependency and the NHA event are shown in Table 2. The highest proportions of NHA among all individuals in the corresponding group occurred for PWD for the disease group hypertension and depression (39.1%, *n* = 482) and for PWOD for the disease group hypertension, diabetes and depression (28.2%, *n* = 320). In all disease groups, PWD spent an average of about 2 years (8 quarters) in their own home after the onset of care-dependency before NHA occurred. PWOD spent between 9.6 quarters (disease group hypertension, diabetes, and heart failure) and 13.5 quarters (disease group hypertension and depression) in their own homes before NHA.

**Table 2:** Number of NHA and average duration until NHA by disease group

Disease group	People with dementia	People without dementia	Total
**Hypertension and diabetes**			
Number of participants in group	2732	6.314	9.046
Number of NHAs (% of individuals in the group)	1018 (37.3%)	1507 (23.9%)	2525 (27.9%)
Quarters until NHA, mean (SD)	8.0 (7.4)	11.6 (9.3)	10.1 (8.8)
**Hypertension and depression**			
Number of participants in group	1231	2006	3237
Number of NHAs (% of individuals in the group)	482 (39.1%)	556 (27.7%)	1038 (32.1%)
Quarters until NHA, mean (SD)	8.9 (8.7)	13.5 (11.1)	11.4 (10.3)
**Hypertension and COPD**			
Number of participants in group	586	1656	2242
Number of NHAs (% of individuals in the group)	175 (29.9%)	319 (19.3%)	494 (22.0%)
Quarters until NHA, mean (SD)	8.1 (7.9)	11.8 (10.3)	10.5 (9.7)
**Hypertension and diabetes and depression**			
Number of participants in group	734	1136	1870
Number of NHAs (% of individuals in the group)	277 (37.7%)	320 (28.2%)	597 (31.9%)
Quarters until NHA, mean (SD)	8.2 (8.3)	11.9 (10.0)	10.2 (9.4)
**Hypertension and diabetes and COPD**			
Number of participants in group	348	1098	1446
Number of NHAs (% of individuals in the group)	96 (27.6%)	184 (16.8%)	280 (19.4%)
Quarters until NHA, mean (SD)	7.9 (6.5)	10.5 (9.0)	9.6 (8.3)
**Hypertension and diabetes and heart failure**			
Number of participants in group	245	627	872
Number of NHAs (% of individuals in the group)	72 (29.4%)	124 (19.8%)	196 (22.5%)
Quarters until NHA, mean (SD)	8.3 (7.4)	9.6 (8.1)	9.1 (7.8)

*NHA* Nursing home admission, *SD* Standard Deviation, *COPD* Chronic obstructive pulmonary disease, *Diabetes* Diabetes mellitus type 2

Additional file 3 shows the HRs and confidence intervals of the multivariable analyses of the variables used to adjust the models. There was a consistent but minor statistically significant increase in the risk for NHA for PWD for age (HRs ranging from 1.03 to 1.05 in four of the six disease groups) and the Charlson Comorbidity Index (HRs ranging from 1.08 to 1.16). Risk for NHA also consistently increased for PWD when they were living alone (HRs ranging from 1.33 to 2.03 in four of the disease groups), had a level of care of 2 (HRs ranging from 1.55 to 2.39 in five of the disease groups), and with a higher number of cognitive impairments while having one functional impairment increased the risk for NHA for PWD and hypertension and diabetes (HR = 3.28, 95% CI 1.43–7.51). For PWOD, age and the Charlson Comorbidity index showed statistically significant associations with an increased risk for NHA for all disease groups, while a risk increase for female sex was shown for PWOD with hypertension and diabetes (HR 1.43, 95% CI 1.26–1.62) and PWOD with hypertension, diabetes and depression (HR 1.97, 95% CI 1.41–2.77). As for PWD, a higher level of care, living alone and a higher number of cognitive impairments also increased the risk of NHA for PWOD throughout the disease groups with the greatest risk for PWOD with hypertension, diabetes and heart failure and a care level of 3 (HR = 3.73, 95% CI 1.83–7.58).

Table 3 shows the HRs as well as the confidence intervals of the multivariable analyses of the influence of individual quality of care on the event of NHA, stratified by disease group for PWD and PWOD.

**Table 3:** Results of the time to event analysis among PWD and PWOD with NHA receiving care for chronic conditions

Disease group	PWD			PWOD		
	Adjusted HR^a^	Lower 95% CI	Upper 95% CI	Adjusted HR^a^	Lower 95% CI	Upper 95% CI
**Hypertension and diabetes**						
Medication for hypertension	1.26	0.97	1.63	1.00	0.82	1.21
Check of HbA1c	0.62*	0.54	0.72	0.54*	0.48	0.61
Ophthalmological examination	0.60*	0.48	0.75	0.59*	0.50	0.69
Fundus examination	0.53*	0.38	0.75	0.45*	0.35	0.58
Acute inpatient treatment of Diabetes	2.67*	1.99	3.60	2.81*	2.28	3.47
Check of triglycerides and cholesterol	0.70*	0.54	0.90	0.70*	0.58	0.84
Check of serum-creatinine	0.72*	0.63	0.83	0.69*	0.62	0.77
Lower-limb amputation	3.10*	1.78	5.55	2.81*	1.94	4.08
**Hypertension and depression**						
Medication for hypertension	1.00	0.57	1.74	0.81	0.50	1.31
Antidepressive medication	0.98	0.80	1.21	1.37*	1.15	1.63
**Hypertension and COPD**						
Medication for hypertension	0.46	0.11	1.94	0.42	0.16	1.14
Inhaled medication	0.54*	0.35	0.84	0.79	0.61	1.02
Non-useful inhaled medication	--^b^	--^b^	--^b^	--^b^	--^b^	--^b^
Acute inpatient treatment of COPD	4.21*	2.20	8.10	2.54*	1.64	3.93
Respiratory therapy	0.86	0.43	1.72	1.15	0.79	1.68
Influenza vaccination	0.64	0.33	1.27	0.79	0.50	1.26
Specific beta-blocker therapy	0.89	0.62	1.29	0.78	0.60	1.01
Specific anticholinergic therapy	0.62	0.34	1.12	1.17	0.88	1.56
Oral corticosteroids	1.40	0.82	2.35	0.86	0.58	1.26
**Hypertension and diabetes and depression**						
Medication for hypertension	0.96	0.54	1.70	0.61	0.32	1.15
Check of HbA1c	0.62*	0.47	0.82	0.49*	0.38	0.62
Ophthalmological examination	0.53*	0.34	0.81	0.57*	0.41	0.80
Fundus examination	0.40*	0.20	0.77	0.56*	0.35	0.89
Acute inpatient treatment of Diabetes	2.12*	1.20	3.74	2.89*	1.82	4.59
Check of triglycerides and cholesterol	0.80	0.52	1.23	0.56*	0.36	0.85
Check of serum-creatinine	0.82	0.63	1.07	0.62*	0.49	0.79
Lower-limb amputation	2.05	0.47	8.88	2.26	0.68	7.59
Antidepressive medication	1.28	0.99	1.66	1.30*	1.02	1.64
**Hypertension and diabetes and COPD**						
Medication for hypertension	1.02	0.35	2.93	0.68	0.29	1.58
Inhaled medication	0.80	0.43	1.50	0.80	0.57	1.11
Non-useful inhaled medication	--^b^	--^b^	--^b^	--^b^	--^b^	--^b^
Acute inpatient treatment of COPD	2.11	0.50	8.85	1.39	0.64	3.00
Respiratory therapy	1.69	0.87	3.27	1.47	0.93	2.33
Influenza vaccination	0.83	0.37	1.84	0.92	0.53	1.60
Specific beta-blocker therapy	0.65	0.49	1.34	0.74	0.54	1.03
Specific anticholinergic therapy	1.16	0.58	2.31	0.82	0.55	1.21
Oral corticosteroids	0.47	0.11	1.96	0.99	0.61	1.60
Check of HbA1c	0.33*	0.19	0.59	0.48*	0.35	0.66
Ophthalmological examination	0.18*	0.06	0.59	0.64*	0.42	0.99
Fundus examination	0.12*	0.02	0.91	0.66	0.38	1.17
Acute inpatient treatment of Diabetes	0.69	0.15	3.17	2.51*	1.26	5.00
Check of triglycerides and cholesterol	0.37	0.13	1.0	0.87	0.55	1.37
Check of serum-creatinine	0.56*	0.34	0.91	0.68*	0.50	0.92
Lower-limb amputation	2.04	0.20	20.97	0.80	0.11	5.82
**Hypertension and diabetes and heart failure**						
Medication for hypertension	0.83	0.23	3.02	0.63	0.27	1.46
Beta-blocker upon heart failure	1.24	0.70	2.17	1.03	0.68	1.57
ACE-inhibitor upon heart failure	0.65	0.36	1.19	1.26	0.82	1.96
Short acting calcium channel blockers	--^b^	--^b^	--^b^	0.00	0.00	0.00
Acute inpatient treatment of heart failure	1.08	0.41	2.89	1.55	0.85	2.83
Check of HbA1c	0.54*	0.30	0.96	0.52*	0.35	0.76
Ophthalmological examination	0.59	0.17	1.38	0.39*	0.20	0.75
Fundus examination	0.43	0.10	1.87	0.33*	0.12	0.89
Acute inpatient treatment of Diabetes	2.65	0.90	7.77	3.19*	1.65	6.16
Check of triglycerides and cholesterol	0.51	0.20	1.31	0.68	0.36	1.26
Check of serum-creatinine	0.56	0.32	1.00	0.53*	0.36	0.78
Lower-limb amputation	1.24	0.19	8.23	2.84	0.91	8.84

*NHA* Nursing home admission, *PWD* People with dementia, *POWD* People without dementia*, HR* Hazard ratio, *CI* Confidence interval, *COPD* Chronic obstructive pulmonary disease, *Diabetes* Diabetes mellitus type 2

^*^Significance level alpha < 0.05

^a^Adjusted for 9 individual-level covariates: age, sex, level of care, direct social network, number of cognitive impairments, number of functional impairments, osteoarthritis, osteoporosis, Charlson comorbidity index

^b^no results determined due to small number of participants in the numerator of the indicator

### Disease group hypertension and diabetes

While drug therapy for hypertension had no significant effect on the length of time to NHA, significant HRs for QI of diabetes care are consistently shown for individuals with hypertension and diabetes, regardless of the presence of dementia. Thereby, protective effects are shown for QI with desirably high values. The likelihood of NHA reduced with regular control of HbA1c levels, ophthalmologic examination, ocular fundus examination, and control of triglycerides, LDL- and HDL-cholesterol, and serum creatinine. Indicators suggesting complications of care, such as emergency inpatient treatment for diabetes (HR = 2.67, 95% CI 1.99–3.60 for PWD and HR = 2.81, 95% CI 2.28–3.47 for PWOD) or lower-limb amputation (HR = 3.10, 95% CI 1.78–5.55 for PWD, HR = 2.81, 95% CI 1.94–4.08 for PWOD) increased the risk of NHA.

### Disease group hypertension and depression

For PWOD, receiving antidepressant pharmacotherapy shortened the time to NHA (HR = 1.37, 95% CI 1.15–1.63), whereas no significant effects were found for PWD. It is noticeable that the non-significant result for PWD indicates a risk reduction contradictory to the risk increase associated with antidepressant pharmacotherapy for PWOD. Drug therapy for hypertension showed no significant association with the risk of NHA in either group.

### Disease group hypertension and COPD

For individuals with hypertension and COPD, receiving emergency inpatient treatment for COPD increased the risk of NHA (HR = 4.21, 95% CI 2.2–8.10 for PWD, HR = 2.54, 95% CI 1.64–3.93 for PWOD). Receiving inhaled medication showed a protective effect for PWD (HR = 0.54, 95% CI 0.35–0.84), while all other QI showed no significant association for this disease group.

### Disease group hypertension, diabetes and depression

For individuals with hypertension, diabetes, and depression, significant effects for the quality of diabetes care were found for PWOD except for lower-limb amputations, following the trend of the association reported above for the disease group hypertension and diabetes. Receiving antidepressant pharmacotherapy significantly increased the likelihood of NHA in PWOD (HR = 1.30, 95% CI 1.02–1.64). In PWD, control of triglycerides, LDL- and HDL-cholesterol, and serum creatinine were no protective factors in this disease group, and the impact of lower-limb amputation was also not significantly associated with the occurrence of NHA. Emergency inpatient treatment for diabetes significantly increased the likelihood of NHA for PWD (HR = 2.12, 95% CI 1.20–3.74).

### Disease group hypertension, diabetes and COPD

The risk-reducing influence of quality of diabetes care, which is significant in other disease groups, is also present in PWD with hypertension and COPD for regular control of the HbA1c-value (HR = 0.33, 95% CI 0.19–0.59), ophthalmologic examination (HR = 0.18, 95% CI 0.06–0.59), ocular fundus examination (HR = 0.12, 95% CI 0.02–0.91), and control of serum creatinine (HR = 0.56, 95% CI 0.34–0.91). In PWOD, significant influences on length of stay in one’s home are shown for control of HbA1c-value (HR = 0.48, 95% CI 0.35–0.66), ophthalmologic examination (HR = 0.64, 95% CI 0.42–0.99), inpatient emergency treatment of diabetes (HR = 2.51, 95% CI 1.26–5.00) and control of serum creatinine (HR = 0.68, 95% CI 0.50–0.92). It is noticeable that the non-significant result for PWD indicates a risk reduction contradictory to the risk increase associated with acute inpatient treatment for PWOD. QI for COPD and hypertension showed no significant associations with the risk of NHA in PWD and PWOD.

### Disease group Hypertension, diabetes and heart failure

Only control of the HbA1c-value (HR = 0.54, 95% CI 0.30–0.96) showed a statistically significant association with the event of NHA in PWD in this disease group. For PWOD, on the other hand, the majority of QI for diabetes care proved to be significant, following the trend evident in other diseases groups of an increase in length of stay in the one’s own home for desirable QI and a reduction in the length of stay for emergency inpatient treatment for diabetes (HR = 3.19, 95% CI 1.65–6.16).

## Discussion

Regardless of belonging to one of the six assessed disease groups, a higher rate of NHA is found for PWD compared to PWOD with a shorter duration until NHA. This finding is consistent with the results of other studies reporting a shorter duration to NHA for PWD compared with PWOD [35] and higher NHA rates depending on the extent of cognitive impairment [36]. The distribution of the included chronic conditions follows reported prevalences in older populations not stratified by care-dependency or dementia as well as prevalences reported in studies on multimorbidity and dementia, with higher proportions of persons with hypertension or diabetes and lower proportions of persons with COPD or heart failure [6, 8, 10, 37].

With an overall rather heterogeneous influence of the QI in the disease groups, QI of diabetes care mostly show consistent associations with the duration of remaining in one’s own home. The initial assumption on the direction of the effect is confirmed for QI of diabetes care. Process indicators targeting following guideline recommendations are associated with remaining in one’s home longer, while indicators assessing complications (inpatient emergency treatment and lower-limp amputation) increase the risk of NHA. It should be noted that the results do not allow a statement on the quality of care for multimorbidity as a whole, as the QI used were originally constructed for single diseases. The care of a single disease may be of higher quality than that of another in the respective disease group. For individuals with and without dementia who have other chronic conditions in addition to diabetes, the quality of diabetes care influences how long they live in their own homes. The presence of diabetes has been repeatedly investigated as a predictor of NHA in other studies and has been both confirmed and found to have a heterogeneous effect, particularly for PWOD [3, 29, 36, 38–40]. Although evidence suggests that PWD with diabetes tend to receive regular eye examinations less frequently than PWOD [12], the results of this study point to the importance of the quality of diabetes care, which shows significant effects for all QI in PWD when hypertension is also present. For QI of care for hypertension, COPD, depression, and heart failure, few significant HRs were found. This parallels prior studies on the predictive validity of theses chronic diseases for which inconclusive results are reported in systematic reviews for depression and hypertension [3, 38], whereas COPD and heart failure do not emerge as significant predictors of NHA [36, 38]. Receipt of antidepressant pharmacotherapy for individuals with hypertension and depression and acute inpatient treatment of diabetes for individuals with hypertension, diabetes and COPD indicate opposing trends regarding the risk increase or reduction of NHA for PWD or PWOD while showing statistically non-significant results for PWD. While the reasons for this remain unknown at this point, this finding makes room for assumptions on the underlying mechanism that might influence the time a person remains in one’s own home and that should be addressed by further research: For PWD, receiving antidepressant pharmacotherapy might result in less frequent behavioural and psychological symptoms of dementia which are relevant factors in the stress perception of informal caregivers and the decision for institutionalization [41]. For PWOD, on the other hand, the receipt of antidepressant pharmacotherapy might be an expression for the severity of a chronic disease that leads to NHA. As for acute inpatient treatment of diabetes, this might be more likely to lead to an adjustment of the home care situation for PWD, such as adding nursing home care services to informal caregiving. The confidence intervals of the reported HRs are often very wide with a small number of events, and thus significant results may be found less frequently in PWD who were overall included in smaller numbers in the observational cohort than PWOD, although individual HRs are significantly above 2, such as the event of emergency inpatient treatment for individuals with diabetes and heart failure and hypertension. Similarly, deficiencies in the diagnosis and documentation of dementia in primary care practices in Germany are known [42], which may result in a mixing of the study groups in real life (PWD could be found among the group labelled as PWOD in this study) cannot be shown through the SHI claims data. In addition, as only diagnoses from 2006 were used to assign people to the disease groups, incident dementia diagnoses as well as other incident diagnoses occurring after 2006 were not included in the analysis. As only a very small number of people assigned to the PWOD group from 2007 onwards had scattered documentation of a dementia diagnosis (up to 5 out of all persons per year for some disease groups) this misclassification does not have a significant impact on the results in terms of the assignment of individuals in the PWD or PWOD group, but needs to be noted when looking at the results.

This study assumes the presence of multimorbidity due to the simultaneous documentation of diagnoses of chronic diseases without knowledge of the individual burden of disease and care trajectory. Irrespective of the individual disease situation, the assumption of multimorbidity enables the examination of the contribution of care processes of chronic diseases to NHA, assuming a fundamental right and entitlement of those in need of care to guideline-compliant primary care. Another approach would be the assumption of comorbidity, i.e. the presence of an index disease to which all other diseases are subordinate in their contribution to the individual burden of disease [4]. For PWD, for example, considering dementia as an index disease can lead to care and treatment decisions being made primarily with regard to the symptoms and progress of dementia. The focus on multimorbidity was chosen because SHI claims data do not contain information on the individually experienced symptoms and burden of disease that would be needed to allow for a weighted assessment of quality aspects. We further referred to the CCM as a framework for cooperative-collaborative design and organization of care delivery that might face particular challenges in the care of PWD who are not always able to communicate their needs and potential shortcomings of care as well as taking on an active patient role. While the CCM provides strong arguments for the utilization of SHI claims data and a population-specific monitoring of care quality, only a small number of outcome indicators were included, as indicators that rely on information on clinical values cannot be calculated from the data used in the study. Indicators aiming at key elements of the CCM, such as communication between patients and healthcare providers, the active role of patients, and the collaboration between providers, would further contribute to the empirical evidence on quality of care for multimorbid PWD and PWOD but respective information is also not included in the underlying data.

Evidence from the literature suggests that, even if often unintentionally, structural barriers impede access to care for PWD, and clinical care pathways or guidelines contain little evidence to support treatment decisions for PWD [12]. However, more adverse QI outcomes and lower utilization of individual care services are generally reported for care-dependent people compared with people without need of care [43, 44]. Knowledge on whether the treatment intensity of chronic diseases changes in favor of other factors, such as quality of life, in the case of care-dependency and dementia, and thus whether guideline-compliant care is provided less frequently for PWD, is scarcely available to date. However, multimorbidity is cited by physicians as a reason to deviate from guidelines or to change their prescribing behaviour [45, 46]. The differences in the average number of prescribed medications and the proportion of individuals with at least one medication from the PRISCUS list to the disadvantage of PWOD in the baseline quarter further indicate differences in prescribing patterns that should be investigated by future research.

In the sense of an ideal-typical design and provision of care according to the CCM, and also taking into account existing empirical evidence on the quality of care for people in need of long-term care, our results point to the importance of targeted population-specific monitoring activities of the quality of primary care and the need for the development of target-group-specific guidelines for treatment decisions considering a need for long-term care, dementia and multimorbidity. In addition, taking responsibility for ensuring the quality of care for multimorbid care-dependent individuals, who are limited in their abilities and possibilities of self-determined participation in the treatment process, differences in care provision could be reduced through a targeted, outreach-oriented design of primary care for PWD and PWOD. In this regard, networking activities and exchange among health care providers and professionals are of particular importance [9], for which experiences and tools from practice and quality networks already are available for utilization.

### Limitations

Although known predictors of NHA are within the range of the QI used in this study and the relevance of the QI is underlined by their relation to German health care guidelines and disease management programs, other factors are known to influence the event of NHA. These factors, which mainly comprise functional and cognitive impairments as well as the structure of the individual direct social network and the experience of stress and caregiver burden in relatives [41, 47, 48], cannot be mapped with SHI claims data. The data linkage with long-term care assessment data allowed for the inclusion of some of these factors for risk adjustment, but data on the direct social network and information needed to calculate the number of cognitive and functional impairments were only available from a single long-term care assessment and were treated as constant over time. Although SHI claims data make it possible to examine large groups of persons [25], our criterion to include at least 200 PWD in the respective disease group did not take into account combinations of diseases that occur increasingly in the context of multimorbidity in populations in need of care that are not stratified according to dementia. This includes chronic pain, chronic renal failure, coronary heart disease, and urinary incontinence [6, 25, 49]. Although individuals were distinct in one of the disease groups, separate models were calculated for each quality indicator. Especially in disease groups that included a large number of quality indicators, the general problem of increasing the probability to get false significant results when conducting multiple tests within the same sample needs to be noted [50]. As our study is of an explorative nature, we did not adjust for multiple testing, which should be a prerequisite for further studies aiming to confirm our exploratory results [50]. Finally, as only disease-specific quality indicators were included in this study, the influence of quality aspects of a more general nature, such as polypharmacy, preventive services or continuity of care remains to be assessed by further research.

## Conclusion

The quality of primary care provided to care-dependent multimorbid people, both with and without dementia, influences whether they remain in their own homes for a longer or shorter period after onset of care-dependency. Health care providers and professionals involved in the ambulatory care of care-dependent multimorbid people should pay special attention to the possibilities and, if necessary, the barriers of guideline-based, coordinated care for chronic illnesses, and evaluate and optimize quality of care on the basis of indicators. This requires the further development of QI that enable population-specific monitoring activities taking the complex constellations of diseases and needs in this population into account. There is future need for research on the contribution of networking and monitoring activities to improving the quality of care and enabling people in need of care to remain in their own homes for as long as possible.

## Supplementary Information

**Additional file 1.**

**Additional file 2.**

**Additional file 3.**

## Data Availability

The data that support the findings of this study are available from the Scientific Institute of the AOK (WIdO) and from the Medical Service of the National Association of Health Insurance Funds (MDS) but restrictions apply to the availability of these data, which were used under license for the current study, and so are not publicly available. Data are however available from the authors upon reasonable request and with permission of the WIdO and the MDS.

## Abbreviations

CCM: Chronic care model
CI: Confidence interval
COPD: Chronic obstructive pulmonary disease
Diabetes: Type 2 diabetes mellitus
HR: Hazard ratios
ICD-10-GM: International Classification of Diseases 10. Revision German Modification
MDK: Medical Service of the Health Funds
MDS: Medical Service of the National Association of Health Insurance Funds
NHA: Nursing home admissions
OLS: Ordinary least squares
PWD: People with dementia
PWOD: People without dementia
QI: Quality indicator/s
SD: Standard deviation
SHI: Statutory health insurance
STROSA: German Reporting Standard for Secondary Data Analyses
