# Correction to: The influence of primary care quality on nursing home admissions in a multimorbid population with and without dementia in Germany: a retrospective cohort study using health insurance claims data

**DOI:** 10.1186/s12877-022-02808-y

**Published:** 2022-02-28

**Authors:** Kathrin Seibert, Susanne Stiefler, Dominik Domhoff, Karin Wolf-Ostermann, Dirk Peschke

**Affiliations:** 1 Faculty 11: Human and Health Sciences, Institute for Public Health and Nursing Research, University of Bremen, Grazer Str. 4, 28359 Bremen, Germany; 2 High Profile Area Health Sciences, University of Bremen, Bremen, Germany; 3 Department of Applied Health Sciences, Hochschule für Gesundheit (University of Applied Sciences), Bochum, Germany

**Correction to: BMC Geriatr 22, 52 (2022)**

**https://doi.org/10.1186/s12877-021-02731-8**

After publication of this article [1], the authors reported an error in the first sentence of results section. In this sentence “5878 PWD” should have read “5876 PWD”.

The original article [1] has been updated.
