# Supplementary material for: The influence of primary care quality on nursing home admissions in a multimorbid population with and without dementia in Germany: a retrospective cohort study using health insurance claims data
Source: BMC Geriatr. 2022 Jan 14;22:52. doi: 10.1186/s12877-021-02731-8 (PMC8759199; doi:10.1186/s12877-021-02731-8)
Supplement: Supplementary file 1 — Additional file 1. [file 12877_2021_2731_MOESM1_ESM.pdf]

**Additional file 1:** Definition of the included indicators of primary care quality and variables

# The influence of primary care quality on nursing home admissions in a multimorbid population with and without dementia in Germany: A retrospective cohort study using health insurance claims data

Kathrin Seibert[1,2], Susanne Stiefler[1,2], Dominik Domhoff[1,2], Karin Wolf-Ostermann[1,2],

Dirk Peschke[1,2,3]

[1] University of Bremen, Faculty 11: Human and Health Sciences, Institute for Public Health and Nursing Research, Germany

[2] University of Bremen, High Profile Area Health Sciences, Germany

[3] Hochschule für Gesundheit (University of Applied Sciences), Department of Applied Health Sciences, Bochum, Germany

*Corresponding Author:*

Kathrin Seibert
kseibert@uni-bremen.de
Grazer Str. 4
28359 Bremen, Germany

## Funding

This research is part of the research project "Nursing Home Admission and its Predictors in Health Care Quality, Living and Assistive Arrangements – a Population-based Cohort Study" [Beginn stationärer Langzeitpflege und seine Prädiktoren in der Versorgungs-, Wohn- und Unterstützungssituation – populationsbasierte Kohortenstudie (Heimeintritt vermeiden)] funded by the German Federal Joint Committee (Gemeinsamer Bundesausschuss, G-BA), grant number 01VSF160

**Additional file 1:** Definition of the included indicators of primary care quality and variables

| Short name of variable or quality indicator | Dimension | Numerator[a] | Denominator[a] | Codes used |
|---|---|---|---|---|
| Asthma: Prevalence | Morbidity, Individual characteristic | Persons with bronchial asthma | All persons | **ICD-10-GM**: J45.- |
| Medication: PRISCUS | Individual characteristic, used to describe sample characteristics | Persons over 65 years with medication from PRISCUS list | Persons over 65 years | **ATC**: C01EB03M01AB01 M01AB51 M02AA23 M02AA73 S01BC01 M01AB11 M01AE03 M02AA10 M01AE53 M01AA01 M02AA01 M01AA51 R05XA10 M01BA01 M01AC01 M02AA07 S01BC06 M01AC06 M01AC56 M01AH05 (NSAID). N02AB02 N02AB52 N02AB72 N02AG03 (opiod analgetics); C08DA81 C01BA01 C01BA51 C01BA71 C01BC04 C07AA07 C07AA57 C07BA07 C01AA05 C01AA55 C01AA02 C01AA52 C01AA08 C01AA58 (antiarrythmic agents); J01XE01 J01XE51 (antibiotics); A03AA A03AB A03CA A03DA A03E N04A R03BB S01FA R03AL (anticholinergic agents); R06AB04 R06AB54 R06AX32 (if year <2015) R06AX07 R06AX57 G04BD04 G04BD04 G04BD07 D04AA14 R06AA04 R06AA54 N05BB01 N05BB51 R06AX33 D04AA13 R01AC09 R06AB03 R01AC08 N05BB52 (antihistamines); B01AC05 B01AC22 (anticoagulants); N06AA09 N06CA01 N06AA12 N06AA02 N06AA04 N06AA21 N06AA06 (antidepressives); N06AB03 (SSRI): N06CA03 N06CA07 N06AF04 (MAO-inhibitors). A04AB02 A04AB52 (antiemetics); C02CA04 G04CA05 C02CA01 C02LE01 C02CA08 G04CA03 (alpha-blockers); C02AC01 N02CX02 N07BB06 S01EA04 C02LC01 C02LC51 C02AA02 N05AX15 N05AX16 C02AA52 C02LA01 C02LA51 C02LA71 C02AB01 C02LB01 C02AB02 (cardio-vascular medication); C08CA05 (calcium-channel blockers); N05AC02 N05AB02 N05AA02 N05AB03 N05AD01 N05AH03 N05AH02 (neuroleptics); N02CA01 N02CA51 N02CA71 C06AA02 C06AA50 N02CA02 N02CA52 N02CA72 N04BC03 N06DX07 N06DX57 (ergotamin and derivates); A06AA01 A06AA51 (laxants); M03BX01 M03BX07 (muscle relexants); N05BA02 N05BA01 N05CD01 N05BA05 N05BA08 N05BA11 N05BA09 N05CD02 N05CD03 N05BA03 (long-acting benzodiazepines); N05BA12 N05CD07 N05CD05 N05BA06 N05BA56 N05BA04 N05CD06 N05CD09 N05CF02 N05CF01 N05CF03 (short- and medium-acting benzodiazepines and z-drugs); N05CM21 R06AA09 A04AB56 R06AA59 A04AB05 D04AA32 N01BX06 N05CM20 R06AA02 S01Gx16 A04Ab55 D04AA82 |

**Additional file 1:** Definition of the included indicators of primary care quality and variables

| Short name of variable or quality indicator | Dimension | Numerator[a] | Denominator[a] | Codes used |
|---|---|---|---|---|
| | | | | N05CX07 R06AA52 (other sedatives); C04AD03 C04AX21 C04AE02 N06DX13 N06BX03 (antidementia, vasodilatatives); N03AA02 N05CA24 (antiepileptics) |
| Medication: Polypharmacy | Individual characteristic, used to describe sample characteristics | Persons over 65 years with at least 5 prescription medications | Persons over 65 years with prescription medication | |
| COPD: Prevalence | Morbidity, individual characteristic | Persons with diagnosed COPD | All persons | **ICD-10-GM**: J43.- J44.- |
| COPD: Inhaled medication | Process indicator | Persons with COPD and inhalative medication | Persons with COPD | **ICD-10-GM**: J43.- J44.-; **ATC**: R03A R03B (Inhalative medication) |
| COPD: Non-useful inhaled medication | Process indicator | Persons with COPD and non-useful inhaled medication | Persons with COPD | **ICD-10-GM: J43.- J44.-; ATC:** R03AK05 |
| COPD: Acute inpatient treatment | Outcome indicator | Persons with acute inpatient treatment of COPD | Persons with COPD | **ICD-10-GM**: J43.- J44.- (hospital discharge diagnosis) |
| COPD: Respiratory therapy | Process indicator | Persons with COPD and respiratory therapy | Persons with COPD | **ICD-10-GM**: J43.- J44.-; **POSNR**: 302-304 501-503 601 6210 6301 6306 (respiratory therapy) |
| COPD: Influenza vaccination | Process indicator | Persons with COPD and influenza vaccination | Persons with COPD | **ICD-10-GM**: J43.- J44.- Z25.1 |
| COPD: Specific beta-blocker therapy | Process indicator | Persons with COPD and specific beta-blocker therapy | Persons with COPD | **ICD-10-GM**: J43.- J44.-; **ATC**: R03AC12 R03AC13 (specific beta blocker) |
| COPD: Specific anticholinergic therapy | Process indicator | Persons with COPD and specific anticholinergic therapy | Persons with COPD | **ICD-10-GM**: J43.- J44.-; **ATC**: R03BB04 (specific anticholinergic) |

**Additional file 1:** Definition of the included indicators of primary care quality and variables

| Short name of variable or quality indicator | Dimension | Numerator[a] | Denominator[a] | Codes used |
|---|---|---|---|---|
| COPD: Oral corticosteroids | Process indicator | Persons with COPD and oral corticosteroids | Persons with COPD | **ICD-10-GM**: J43.- J44.-; **ATC**: H02AB01 H02AB02 H02AB03 H02AB04 H02AB05 H02AB06 H02AB07 H02AB08 H02AB09 (corticosteroids) |
| Hypertension: Prevalence | Morbidity, individual characteristic | Persons with hypertension | All persons | **ICD-10-GM**: I10.- I11.- I12.- I13.- I15.- |
| Hypertension: Medication for hypertension | Process indicator | Persons with hypertension and pharmacotherapy | Persons with hypertension | **ICD-10-GM**: I10.- I11.- I12.- I13.- I15.- **ATC**: C02 (antihypertensives) |
| Heart failure: Prevalence | Morbidity, individual characteristic | Persons with heart failure | All persons | **ICD-10-GM**: I50.1 |
| Heart failure: ACE-inhibitor upon heart failure | Process indicator | Persons with heart failure treated with ACE-inhibitor or AT1-blocker | Persons with heart failure | **ICD-10-GM**: I50.1; **ATC**: C09 |
| Heart failure: Beta-blocker upon heart failure | Process indicator | Persons with heart failure and beta-blocker | Persons with heart failure | **ICD-10-GM**: I50.1; **ATC**: C07 |
| Heart failure: Short acting calcium channel blockers upon heart failure | Process indicator | Persons with heart failure and short acting calcium channel blockers | Persons with heart failure | **ICD-10-GM**: I50.1; **ATC**: C08CA05 |
| Heart failure: Acute inpatient treatment of heart failure | Outcome indicator | Persons with heart failure and acute inpatient treatment of heart failure | Persons with heart failure | **ICD-10-GM**: I50.1 (hospital discharge diagnosis) |
| Dementia: Prevalence | Morbidity, individual characteristic | Persons with dementia | All persons | **ICD-10-GM**: F00.- F01.- F02.- F03.- |
| T2D: Prevalence | Morbidity, individual characteristic | Persons with type 2 diabetes mellitus | All persons | **ICD-10-GM**: E11.- |

**Additional file 1:** Definition of the included indicators of primary care quality and variables

| Short name of variable or quality indicator | Dimension | Numerator[a] | Denominator[a] | Codes used |
|---|---|---|---|---|
| T2D: HbA1c | Process indicator | Persons with type 2 diabetes mellitus and check of HbA1c | Persons with type 2 diabetes mellitus | **ICD-10-GM**: E11.-; **GONR**:32094 90321 90310 90310A (HbA1c check) |
| T2D: Ophthalmological examination | Process indicator | Persons with type 2 diabetes mellitus and ophthalmological examination | Persons with type 2 diabetes mellitus | **ICD-10-GM**: E11.- **GONR**: 06210 06211 06212 (ophthalmological examination) |
| T2D: Fundus examination | Process indicator | Persons with type 2 diabetes mellitus and fundus examination | Persons with type 2 diabetes mellitus | **ICD-10-GM**: E11.-; **GONR**: 06331 06333 (fundus examination) |
| T2D: Triglycerides and cholesterol | Process indicator | Persons with type 2 diabetes mellitus and check of triglycerides, LDL- and HDL-cholesterol | Persons with type 2 diabetes mellitus | **ICD-10-GM**: E11.-; **GONR**: 32060 32061 32062 32063 (laboratory examinations) |
| T2D: Serum-creatinine | Process indicator | Persons with type 2 diabetes mellitus and check of serum creatinine | Persons with type 2 diabetes mellitus | **ICD-10-GM**: E11.-; **GONR**: 32066 32067 |
| T2D: Acute inpatient treatment of T2D | Outcome indicator | Persons with diabetes mellitus and acute inpatient treatment of diabetes mellitus | Persons with diabetes mellitus | **ICD-10-GM**: E11.- (hospital discharge diagnosis) |
| TD2: Lower-limb amputation | Outcome indicator | Persons with type 2 diabetes mellitus and lower-limb amputation | Persons with type 2 diabetes mellitus | **ICD-10-GM**: E11.-; **OPS:** 5864 58690 5865 |
| Osteoarthritis: Prevalence | Morbidity, individual characteristic | Persons with osteoarthritis | All persons | **ICD-10-GM**: M15.- M16.- M17.- M18.- M19.- |
| Osteoporosis: Prevalence | Morbidity, individual characteristic | Persons with osteoporosis | All persons | **ICD-10-GM**: M80.- M81.- M82.- |

**Additional file 1:** Definition of the included indicators of primary care quality and variables

| **Short name of variable or quality indicator** | **Dimension** | **Numerator[a]** | **Denominator[a]** | **Codes used** |
|---|---|---|---|---|
| Depression: Prevalence | Morbidity, individual characteristic | Persons with diagnosed depression | All persons | **ICD-10-GM**: F32.- F33.- F34.1 |
| Depression: Anti-depressive pharmacotherapy | Process indicator | Persons with diagnosed depression and anti-depressive pharmacotherapy | All persons with diagnosed depression | **ICD-10-GM**: F32.- F33.- F34.1; **ATC**: N06AA N06AB N06AF N06AG N06AX N05AN01 (anti-depressive pharmacotherapy) |
| Charlson comorbidity index | Individual characteristic | | | Codes according to Quan, H., et al., Coding algorithms for defining comorbidities in ICD-9-CM and ICD-10 administrative data. Med Care, 2005. 43(11): p. 1130-9. |
| Age in years | Individual characteristic | | | |
| Sex female (reference male) | Individual characteristic | | | |
| Level of care none (reference level of care 1) | Individual characteristic | | | |
| Level of care 2 (reference level of care 1) | Individual characteristic | | | |
| Level of care 3 (reference level of care 1) | Individual characteristic | | | |
| Number of prescribed drugs | Individual characteristic | | | |
| Social network living alone | Individual characteristic | | | |

**a)** Flagged with 1 or 0 for fulfilling or not fulfilling the respective numerator or denominator condition in the dataset.
**ICD-10-GM**: International Statistical Classification of Diseases and Related Health Problems, 10$^{th}$ revision, German modification
**ATC**: Anatomical Therapeutic Chemical Classification System
**OPS**: German Operation and Procedure Classification System; official coding system for medical procedures in German hospitals
**GONR**: Item number (Gebührenordnungsposition) from the German Doctor's fee scale (Einheitlicher Bewertungsmaßstab, EBM)
