# Supplementary material for: The influence of primary care quality on nursing home admissions in a multimorbid population with and without dementia in Germany: a retrospective cohort study using health insurance claims data
Source: BMC Geriatr. 2022 Jan 14;22:52. doi: 10.1186/s12877-021-02731-8 (PMC8759199; doi:10.1186/s12877-021-02731-8)
Supplement: Supplementary file 2 — Additional file 2. [file 12877_2021_2731_MOESM2_ESM.pdf]

Additional file 2: Baseline results of quality indicators of primary care for chronic conditions for multimorbid people with and without dementia, 2007 1st quarter

The influence of primary care quality on nursing home admissions in a multimorbid population with and without dementia in Germany: A retrospective cohort study using health insurance claims data

Kathrin Seibert[1,2], Susanne Stiefler[1,2], Dominik Domhoff[1,2], Karin Wolf-Ostermann[1,2], Dirk Peschke[1,2,3]

[1] University of Bremen, Faculty 11: Human and Health Sciences, Institute for Public Health and Nursing Research, Germany

[2] University of Bremen, High Profile Area Health Sciences, Germany

[3] Hochschule für Gesundheit (University of Applied Sciences), Department of Applied Health Sciences, Bochum, Germany

Corresponding Author:

Kathrin Seibert
kseibert@uni-bremen.de
Grazer Str. 4
28359 Bremen, Germany

Funding

This research is part of the research project "Nursing Home Admission and its Predictors in Health Care Quality, Living and Assistive Arrangements – a Population-based Cohort Study" [Beginn stationärer Langzeitpflege und seine Prädiktoren in der Versorgungs-, Wohn- und Unterstützungssituation – populationsbasierte Kohortenstudie (Heimeintritt vermeiden)] funded by the German Federal Joint Committee (Gemeinsamer Bundesausschuss, G-BA), grant number 01VSF16042.

Additional file 2: Baseline results of quality indicators of primary care for chronic conditions for multimorbid people with and without dementia,
2007 1st quarter

Disease group hypertension and diabetes

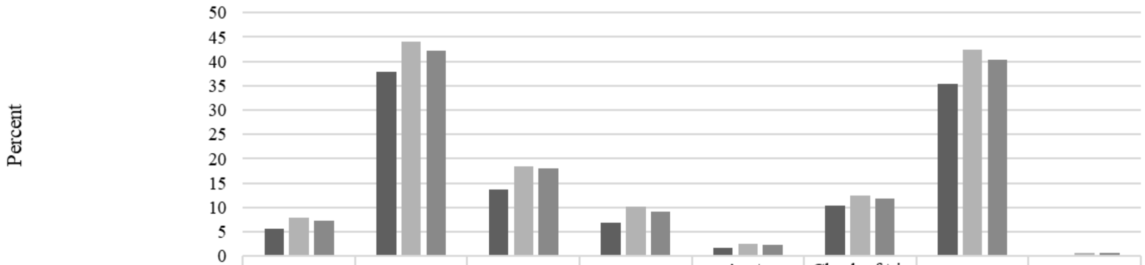

	Medication for hyper-tension	Check of HbA1c	Ophthal-mological exam-ination	Fundus exam-ination	Acute inpatient treatment of diabetes	Check of tri-glycerides and cholesterol	Check of serum-creatinine	Lower-limb amputation
with dementia (n=2732)	5.64	37.85	13.58	6.92	1.76	10.40	35.40	0.26
without dementia (n=6314)	7.84	44.04	18.53	10.14	2.61	12.34	42.46	0.74
total (n=9046)	7.17	42.17	17.94	9.16	2.35	11.75	40.33	0.60

Disease group hypertension and depression

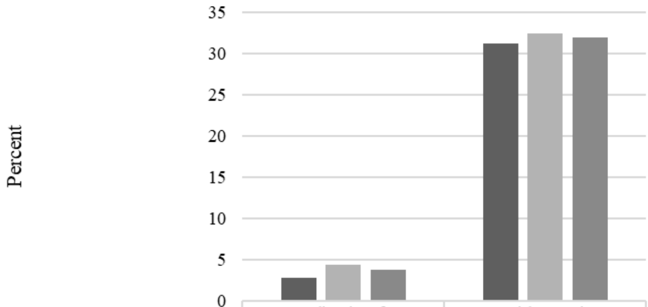

	Medication for hypertension	Antidepressive medication
with dementia (n=1231)	2.76	31.19
without dementia (n=2006)	4.34	32.5
total (n=3237)	3.74	32

Additional file 2: Baseline results of quality indicators of primary care for chronic conditions for multimorbid people with and without dementia,
2007 1st quarter

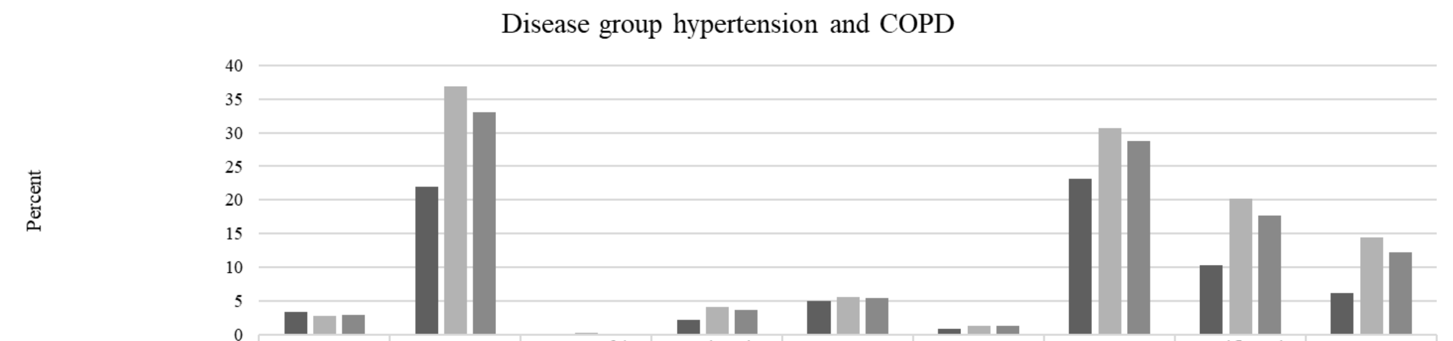

	Medication for hypertension	Inhaled medication	Non-useful inhaled medication	Acute inpatient treatment of COPD	Respiratory therapy	Influenza vaccination	Specific beta-blocker therapy	Specific anti-cholinergic therapy	Oral cortico-steroids
with dementia (n=586)	3.41	22.01	0	2.22	4.95	0.85	23.21	10.24	6.14
without dementia (n=1656)	2.72	36.9	0.18	4.05	5.62	1.33	30.68	20.23	14.43
total (n=2242)	2.9	33.01	0.13	3.57	5.44	1.2	28.72	17.62	12.27

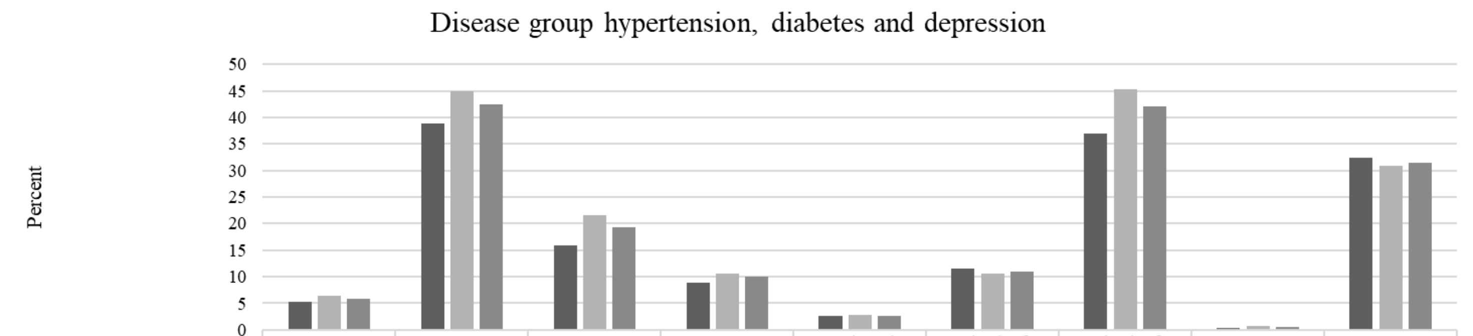

	Medication for hypertension	Check of HbA1c	Ophthalmological examination	Fundus examination	Acute inpatient treatment of Diabetes	Check of triglycerides and cholesterol	Check of serum-creatinine	Lower-limb amputation	Antidepressive medication
with dementia (n=734)	5.18	38.83	15.8	8.86	2.59	11.44	36.92	0.27	32.43
without dementia (n=1136)	6.34	44.89	21.65	10.65	2.73	10.65	45.25	0.7	30.81
total (n=1870)	5.88	42.51	19.36	9.95	2.67	10.96	41.98	0.53	31.44

Additional file 2: Baseline results of quality indicators of primary care for chronic conditions for multimorbid people with and without dementia,
2007 1st quarter

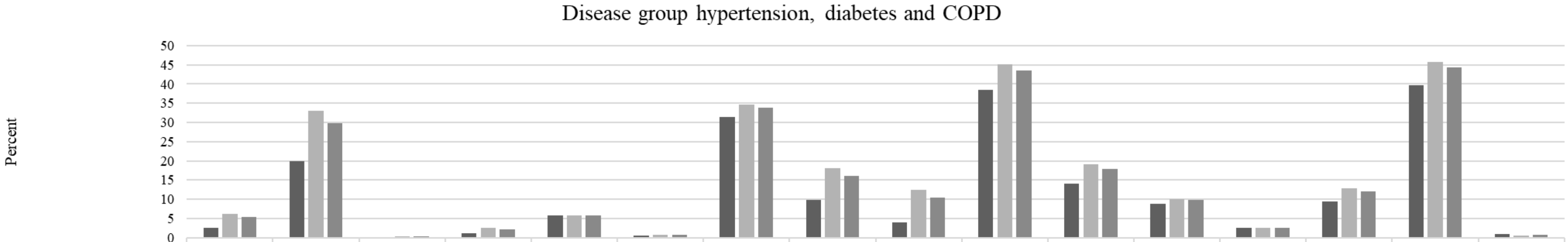

	Medication for hypertension	Inhaled medication	Non-useful inhaled medication	Acute inpatient treatment of COPD	Respiratory therapy	Influenza vaccination	Specific beta-blocker therapy	Specific anticholinergic therapy	Oral corticosteroids	Check of HbA1c	Ophthalmological examination	Fundus examination	Acute inpatient treatment of Diabetes	Check of triglycerides and cholesterol	Check of serum-creatinine	Lower-limb amputation
with dementia (n=348)	2.59	19.83	0	1.15	5.75	0.57	31.32	9.77	4.02	38.51	14.08	8.91	2.59	9.48	39.66	0.86
without dementia (n=1098)	6.19	32.97	0.36	2.46	5.74	0.73	34.61	18.03	12.39	45.08	19.03	10.11	2.46	12.84	45.81	0.64
total (n=1446)	5.33	29.81	0.28	2.14	5.74	0.69	33.82	16.04	10.37	43.5	17.84	9.82	2.49	12.03	44.33	0.69

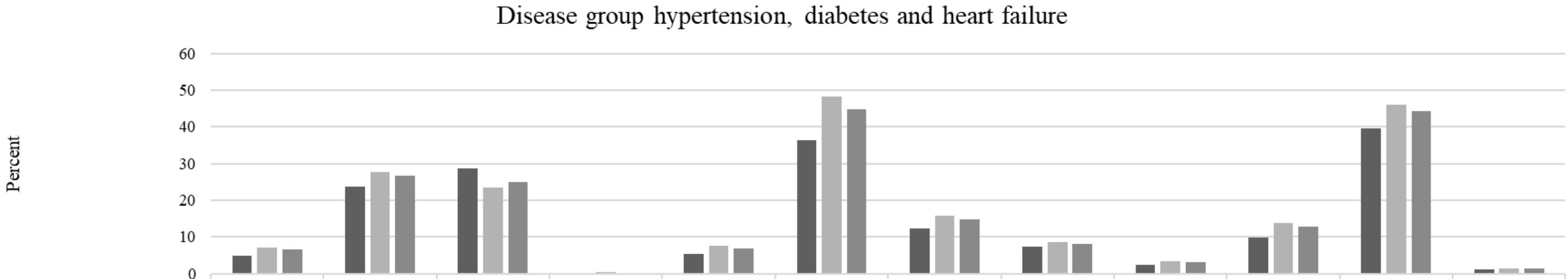

	Medication for hypertension	Beta-blocker upon heart failure	ACE-inhibitor upon heart failure	Short acting calcium channel blockers	Acute inpatient treatment of heart failure	Check of HbA1c	Ophthalmological examination	Fundus examination	Acute inpatient treatment of Diabetes	Check of triglycerides and cholesterol	Check of serum-creatinine	Lower-limb amputation
with dementia (n=245)	4.9	23.67	28.57	0	5.31	36.33	12.24	7.35	2.45	9.8	39.59	1.22
without dementia (n=627)	7.18	27.75	23.44	0.32	7.5	48.17	15.79	8.45	3.35	13.88	46.09	1.28
total (n=872)	6.54	26.61	24.89	0.23	6.88	44.84	14.79	8.14	3.1	12.73	44.27	1.26
