# Supplementary material for: The influence of primary care quality on nursing home admissions in a multimorbid population with and without dementia in Germany: a retrospective cohort study using health insurance claims data
Source: BMC Geriatr. 2022 Jan 14;22:52. doi: 10.1186/s12877-021-02731-8 (PMC8759199; doi:10.1186/s12877-021-02731-8)
Supplement: Supplementary file 3 — Additional file 3. [file 12877_2021_2731_MOESM3_ESM.pdf]

Additional file 3: Coefficients for age, sex, level of care, social network, number of cognitive impairments, number of functional impairments, comorbidity index, osteoarthritis and osteoporosis among PWD and PWOD with NHA.

The influence of primary care quality on nursing home admissions in a multimorbid population with and without dementia in Germany: A retrospective cohort study using health insurance claims data

Kathrin Seibert[1,2], Susanne Stiefler[1,2], Dominik Domhoff[1,2], Karin Wolf-Ostermann[1,2],

Dirk Peschke[1,2,3]

[1] University of Bremen, Faculty 11: Human and Health Sciences, Institute for Public Health and Nursing Research, Germany

[2] University of Bremen, High Profile Area Health Sciences, Germany

[3] Hochschule für Gesundheit (University of Applied Sciences), Department of Applied Health Sciences, Bochum, Germany

Corresponding Author:

Kathrin Seibert
kseibert@uni-bremen.de
Grazer Str. 4
28359 Bremen, Germany

Funding

This research is part of the research project "Nursing Home Admission and its Predictors in Health Care Quality, Living and Assistive Arrangements – a Population-based Cohort Study" [Beginn stationärer Langzeitpflege und seine Prädiktoren in der Versorgungs-, Wohn- und Unterstützungssituation – populationsbasierte Kohortenstudie (Heimeintritt vermeiden)] funded by the German Federal Joint Committee (Gemeinsamer Bundesausschuss, G-BA), grant number 01VSF16042.

Additional file 3: Coefficients for age, sex, level of care, social network, number of cognitive impairments, number of functional impairments, comorbidity index, osteoarthritis and osteoporosis among PWD and PWOD with NHA.

	PWD			PWOD		
**Disease group**	**HR**	**Lower 95% CI**	**Upper 95% CI**	**HR**	**Lower 95% CI**	**Upper 95% CI**
**Hypertension and diabetes**						
Age	1.03*	1.02	1.04	1.06*	1.05	1.07
Female sex	1.20	1.04	1.40	1.43*	1.26	1.62
Level of care[a]: 2	1.91*	1.65	2.20	2.27*	2.01	2.55
Level of care: 3	1.70*	1.35	2.15	2.10*	1.69	2.60
Level of care: none	0.20	0.05	0.78	0.19*	0.07	0.50
Social network[a]: living alone	1.60*	1.40	1.83	1.88*	1.69	2.10
Number of cognitive impairments[a]: 1	1.07	0.77	1.49	1.11	0.93	1.33
Number of cognitive impairments: 2	0.90	0.62	1.32	1.26	1.03	1.54
Number of cognitive impairments: 3	1.32	0.95	1.84	1.11	0.88	1.40
Number of cognitive impairments: 4	1.24	0.91	1.68	1.24	0.98	1.58
Number of cognitive impairments: 5	1.13	0.84	1.52	1.15	0.90	1.47
Number of cognitive impairments: 6	1.59*	1.25	2.01	1.56*	1.25	1.96
Number of cognitive impairments: 7	1.35*	1.09	1.67	1.89*	1.55	2.31
Number of cognitive impairments: 8	1.36*	1.10	1.68	1.79*	1.44	2.23
Number of cognitive impairments: 9	1.64*	1.31	2.04	1.18	0.91	1.51
Number of functional impairments[a]: 1	3.28*	1.43	7.51	1.68	0.88	3.18
Number of functional impairments: 2	1.61	0.92	2.83	1.18	0.76	1.84
Number of functional impairments: 3	1.75	1.05	2.92	1.26	0.84	1.89
Number of functional impairments: 4	1.18	0.71	1.95	1.04	0.69	1.55
Charlson Comorbidity Index	1.08*	1.06	1.10	1.19*	1.17	1.21
Osteoarthritis	0.87*	0.75	1.00	0.95	0.86	1.06
Osteoporosis	1.09	0.90	1.31	0.97	0.85	1.11
**Hypertension and depression**						
Age	1,01	0,99	1,02	1,05*	1,04	1,06
Female sex	1,00	0,79	1,28	1,41	1,08	1,85
Level of care: 2	1,55*	1,25	1,92	2,06*	1,70	2,51
Level of care: 3	1,10	0,78	1,56	2,02*	1,43	2,84
Level of care: none	--[b)]	--[b)]	--[b)]	0,23*	0,06	0,92
Social network: living alone	1,54*	1,28	1,85	1,61*	1,33	1,95
Number of cognitive impairments: 1	0,85	0,52	1,40	0,95	0,69	1,30

Additional file 3: Coefficients for age, sex, level of care, social network, number of cognitive impairments, number of functional impairments, comorbidity index, osteoarthritis and osteoporosis among PWD and PWOD with NHA.

	PWD			PWOD		
**Disease group**	**HR**	**Lower 95% CI**	**Upper 95% CI**	**HR**	**Lower 95% CI**	**Upper 95% CI**
Number of cognitive impairments: 2	1,35	0,87	2,09	1,51*	1,12	2,06
Number of cognitive impairments: 3	1,03	0,61	1,74	1,66*	1,19	2,30
Number of cognitive impairments: 4	0,97	0,59	1,58	1,21	0,80	1,83
Number of cognitive impairments: 5	1,41	0,96	2,08	1,40	0,96	2,04
Number of cognitive impairments: 6	1,39	0,97	1,98	1,56*	1,06	2,31
Number of cognitive impairments: 7	1,10	0,79	1,54	1,99*	1,41	2,80
Number of cognitive impairments: 8	1,17	0,86	1,59	1,48*	1,05	2,09
Number of cognitive impairments: 9	1,48*	1,08	2,03	1,45	0,98	2,16
Number of functional impairments: 1	0,75	0,21	2,69	0,98	0,28	3,36
Number of functional impairments: 2	1,36	0,73	2,51	1,08	0,63	1,88
Number of functional impairments: 3	0,80	0,45	1,41	0,88	0,53	1,44
Number of functional impairments: 4	0,71	0,41	1,22	0,80	0,49	1,29
Charlson Comorbidity Index	1,12*	1,09	1,16	1,16*	1,13	1,19
Osteoarthritis	0,77*	0,63	0,94	0,91	0,76	1,09
Osteoporosis	1,29*	1,06	1,59	1,23*	1,02	1,48
**Hypertension and COPD**						
Age	1,05*	1,03	1,08	1,05*	1,03	1,07
Female sex	1,11	0,77	1,61	1,19	0,91	1,55
Level of care: 2	2,06*	1,48	2,87	2,05*	1,60	2,63
Level of care: 3	1,68	0,93	3,04	1,60	0,97	2,63
Level of care: none	1,57	0,21	11,60	0,42	0,10	1,70
Social network: living alone	1,27	0,91	1,78	1,68*	1,29	2,19
Number of cognitive impairments: 1	1,68	0,84	3,38	1,01	0,69	1,49
Number of cognitive impairments: 2	2,08	0,92	4,75	0,91	0,59	1,41
Number of cognitive impairments: 3	1,17	0,49	2,80	0,58	0,30	1,13
Number of cognitive impairments: 4	1,57	0,72	3,40	1,27	0,74	2,19
Number of cognitive impairments: 5	1,72	0,85	3,46	1,59	0,88	2,88
Number of cognitive impairments: 6	2,73*	1,43	5,20	0,91	0,52	1,61
Number of cognitive impairments: 7	1,13	0,61	2,07	2,07*	1,21	3,55
Number of cognitive impairments: 8	1,59	0,96	2,65	1,58	0,96	2,61
Number of cognitive impairments: 9	1,98*	1,14	3,41	1,06	0,55	2,03
Number of functional impairments: 1	--[b)]	--[b)]	--[b)]	2,56	0,76	8,67
Number of functional impairments: 2	1,02	0,33	3,13	1,88	0,84	4,20

Additional file 3: Coefficients for age, sex, level of care, social network, number of cognitive impairments, number of functional impairments, comorbidity index, osteoarthritis and osteoporosis among PWD and PWOD with NHA.

	PWD			PWOD		
**Disease group**	**HR**	**Lower 95% CI**	**Upper 95% CI**	**HR**	**Lower 95% CI**	**Upper 95% CI**
Number of functional impairments: 3	0,60	0,23	1,61	1,25	0,60	2,63
Number of functional impairments: 4	0,63	0,25	1,59	1,12	0,54	2,33
Charlson Comorbidity Index	1,07*	1,00	1,15	1,16*	1,12	1,20
Osteoarthritis	1,19	0,86	1,64	1,00	0,80	1,26
Osteoporosis	0,91	0,61	1,36	1,11	0,86	1,44
**Hypertension and diabetes and depression**						
Age	1,04*	1,02	1,06	1,04*	1,02	1,06
Female sex	1,19	0,87	1,63	1,97*	1,41	2,77
Level of care: 2	1,98*	1,52	2,58	1,91*	1,49	2,46
Level of care: 3	1,37	0,85	2,21	1,61	0,94	2,75
Level of care: none	1,75	0,41	7,47	0,46	0,14	1,51
Social network: living alone	1,33*	1,03	1,70	1,53*	1,21	1,94
Number of cognitive impairments: 1	2,17*	1,31	3,59	1,23	0,81	1,86
Number of cognitive impairments: 2	0,92	0,46	1,82	1,33	0,87	2,04
Number of cognitive impairments: 3	2,01*	1,04	3,88	1,40	0,86	2,27
Number of cognitive impairments: 4	1,64	0,89	3,02	1,34	0,82	2,18
Number of cognitive impairments: 5	2,91*	1,68	5,04	2,38*	1,52	3,71
Number of cognitive impairments: 6	1,14	0,66	1,99	1,71*	1,06	2,75
Number of cognitive impairments: 7	1,75*	1,14	2,70	1,64*	1,02	2,64
Number of cognitive impairments: 8	1,74*	1,15	2,63	2,00*	1,29	3,10
Number of cognitive impairments: 9	1,71*	1,09	2,68	1,29	0,72	2,31
Number of functional impairments: 1	0,99	0,19	5,04	2,04	0,61	6,78
Number of functional impairments: 2	1,92	0,76	4,84	0,66	0,25	1,73
Number of functional impairments: 3	1,37	0,58	3,21	0,89	0,38	2,08
Number of functional impairments: 4	0,94	0,41	2,17	0,65	0,28	1,51
Charlson Comorbidity Index	1,15*	1,11	1,20	1,18*	1,14	1,22
Osteoarthritis	0,83	0,64	1,08	0,82	0,65	1,04
Osteoporosis	0,93	0,69	1,25	1,12	0,85	1,47
**Hypertension and diabetes and COPD**						
Age	1,04	1,01	1,07	1,05*	1,03	1,08
Female sex	1,01	0,61	1,67	0,97	0,71	1,32
Level of care: 2	2,39*	1,51	3,76	2,05*	1,46	2,88
Level of care: 3	2,10	0,83	5,31	0,90	0,37	2,19

Additional file 3: Coefficients for age, sex, level of care, social network, number of cognitive impairments, number of functional impairments, comorbidity index, osteoarthritis and osteoporosis among PWD and PWOD with NHA.

	PWD			PWOD		
**Disease group**	**HR**	**Lower 95% CI**	**Upper 95% CI**	**HR**	**Lower 95% CI**	**Upper 95% CI**
Level of care: none	1,31	0,15	11,72	0,51	0,07	3,74
Social network: living alone	2,03*	1,29	3,21	2,37*	1,73	3,25
Number of cognitive impairments: 1	0,82	0,18	3,70	1,39	0,88	2,20
Number of cognitive impairments: 2	0,52	0,15	1,83	0,59	0,31	1,16
Number of cognitive impairments: 3	0,97	0,31	3,01	0,84	0,38	1,83
Number of cognitive impairments: 4	2,06	0,88	4,87	0,59	0,26	1,36
Number of cognitive impairments: 5	2,08	0,89	4,86	0,50	0,18	1,41
Number of cognitive impairments: 6	1,49	0,59	3,78	1,71	0,87	3,35
Number of cognitive impairments: 7	1,94	0,87	4,30	1,94	0,95	3,97
Number of cognitive impairments: 8	2,38*	1,13	5,01	2,49*	1,42	4,39
Number of cognitive impairments: 9	2,06	1,00	4,28	1,23	0,45	3,42
Number of functional impairments: 1	--[b)]	--[b)]	--[b)]	--[b)]	--[b)]	--[b)]
Number of functional impairments: 2	3,29	0,33	32,87	3,97	0,90	17,50
Number of functional impairments: 3	3,66	0,45	29,67	2,68	0,64	11,17
Number of functional impairments: 4	2,34	0,29	18,66	2,40	0,59	9,82
Charlson Comorbidity Index	1,10*	1,02	1,18	1,17*	1,12	1,23
Osteoarthritis	1,18	0,76	1,82	0,76	0,56	1,05
Osteoporosis	1,15	0,63	2,10	1,46	1,02	2,08
**Hypertension and diabetes and heart failure**						
Age	1,06*	1,02	1,10	1,05*	1,02	1,08
Female sex	0,81	0,43	1,52	1,06	0,67	1,68
Level of care: 2	1,69	0,92	3,11	2,07*	1,38	3,10
Level of care: 3	1,36	0,51	3,62	3,73*	1,83	7,58
Level of care: none	--[b)]	--[b)]	--[b)]	--[b)]	--[b)]	--[b)]
Social network: living alone	1,59	0,94	2,71	2,22*	1,48	3,33
Number of cognitive impairments: 1	1,34	0,43	4,18	0,84	0,41	1,71
Number of cognitive impairments: 2	1,08	0,29	3,98	1,02	0,49	2,11
Number of cognitive impairments: 3	1,06	0,34	3,32	1,54	0,79	2,98
Number of cognitive impairments: 4	2,02	0,45	9,08	0,92	0,32	2,61
Number of cognitive impairments: 5	1,93	0,73	5,14	0,61	0,24	1,59
Number of cognitive impairments: 6	1,43	0,47	4,39	0,75	0,32	1,76
Number of cognitive impairments: 7	1,27	0,55	2,94	1,79	0,81	3,94
Number of cognitive impairments: 8	1,68	0,69	4,06	1,25	0,55	2,85

Additional file 3: Coefficients for age, sex, level of care, social network, number of cognitive impairments, number of functional impairments, comorbidity index, osteoarthritis and osteoporosis among PWD and PWOD with NHA.

	PWD			PWOD		
**Disease group**	**HR**	**Lower 95% CI**	**Upper 95% CI**	**HR**	**Lower 95% CI**	**Upper 95% CI**
Number of cognitive impairments: 9	1,10	0,37	3,33	0,61	0,15	2,50
Number of functional impairments: 1	--[b)]	--[b)]	--[b)]	0,62	0,04	10,69
Number of functional impairments: 2	1,73	0,13	22,34	1,07	0,10	11,03
Number of functional impairments: 3	1,61	0,17	15,32	1,38	0,17	11,36
Number of functional impairments: 4	0,84	0,09	8,22	1,05	0,13	8,66
Charlson Comorbidity Index	1,16*	1,08	1,26	1,15*	1,08	1,21
Osteoarthritis	0,66	0,38	1,17	0,88	0,59	1,29
Osteoporosis	2,13	0,98	4,65	1,23	0,78	1,95

[a)] Reference for level of care: 1, reference for social network: not living alone, reference for number of cognitive impairments: 0, reference for number of functional impairments: 0. [b)] no results determined due to small number or no individuals with the respective characteristic. [*)] Significance level alpha <0.05. *NHA* Nursing home admission, *PWD* People with dementia, *POWD* People without dementia, *HR* Hazard ratio. *CI* Confidence interval. *COPD* Chronic obstructive pulmonary disease. *Diabetes* Diabetes mellitus type 2.
